# Sleep, autonomic function, and cognition in the U.S. POINTER trial

**DOI:** 10.1002/alz.092320

**Published:** 2025-01-09

**Authors:** Hossam A Shaltout, Kathleen M. Hayden, Katelyn R Garcia, Xiaoyan Leng, Marjorie Howard, Margaret Scales, Margaret C. Culkin, Laura D Baker, Heather M Snyder, Kathryn V Papp, Margie J Bailey, Tina E Brinkley

**Affiliations:** ^1^ Wake Forest University School of Medicine, Winston‐Salem, NC USA; ^2^ Wake Forest University School of Medicine, Winston Salem, NC USA; ^3^ Wake Forest University, Winston‐Salem, NC USA; ^4^ Alzheimer’s Association, Chicago, IL USA; ^5^ Massachusetts General Hospital, Brigham and Women’s Hospital, Harvard Medical School, Boston, MA USA

## Abstract

**Background:**

Sleep disorders have been associated with cognitive impairment and dementia risk. Measures of autonomic function including baroreflex sensitivity (BRS) and heart rate variability (HRV) have also been associated with sleep quality. The extent to which sleep disorders are linked to autonomic function and alter the risk of Alzheimer’s disease (AD) in older adults remains unclear. We investigated the association between sleep measures and autonomic function measures in 193 participants enrolled in the U.S. POINTER trial (mean age: 67.8±5.4 years, 65% female, 35% people of color).

**Method:**

Continuous blood pressure and ECG recordings were used to assess baroreflex sensitivity (BRS), blood pressure variability (BPV), HRV measured as root mean square of successive differences (rMSSD) and standard deviation of beat to beat interval (SDNN), and sympathovagal balance (LF/HF ratio). Sleep quality was assessed by multiple measures including the apnea‐hypopnea index (AHI), oxygen desaturation index (ODI), sleep duration, sleep efficiency, and wake after sleep onset (WASO). Linear regression was used to examine cross‐sectional associations between the outcomes of interest adjusting for age, sex, race/ethnicity, education, and site.

**Result:**

The majority of participants had normal sleep breathing pattern, with AHI and ODI ≤15 (n = 154, 80.0%). Individuals with AHI or ODI >15 have lower BRS (p<0.025), lower SDNN (p<0.026) and slightly higher LF/HF ratio and BPV (p = 0.09). BRS was positively associated with sleep duration (p = 0.04), high frequency alpha (p = 0.039), and sleep efficiency (p = 0.005), as well as global cognition (p = 0.038), episodic memory (p = 0.004), and mini‐mental state examination (MMSE) score (p = 0.06). BRS was also negatively correlated with WASO (p = 0.011). There was also a positive association between SDNN and 3MSE (p = 0.025), with similar trends for the association between rMSSD and both global cognition and processing speed (p = 0.08). Adjusting for AHI or ODI score strengthened the associations between autonomic function and cognition.

**Conclusion:**

In this cohort of older adults, measures of autonomic function were inversely correlated with sleep disorders and positively correlated with cognitive function. Sleep disorders are linked to alterations in autonomic function, which are likely to exert significant downstream effects on cardiovascular health and brain function. These effects may potentially influence the development of AD.

